# Complexity and Statistical Physics Approaches to Earthquakes

**DOI:** 10.3390/e26010059

**Published:** 2024-01-10

**Authors:** Georgios Michas

**Affiliations:** Section of Geophysics-Geothermics, Department of Geology and Geoenvironment, National and Kapodistrian University of Athens, 15772 Athens, Greece; gemichas@geol.uoa.gr

This Special Issue of *Entropy*, “Complexity and Statistical Physics Approaches to Earthquakes”, sees the successful publication of 11 original scientific articles. This collection presents broad perspectives on the complexity of earthquakes and the use of statistical physics as a consistent, but also necessary, theoretical framework to unravel the complex dynamics that lead to the nucleation and evolution of the phenomenon. 

Earthquakes are inherently a complex phenomenon, incorporating intermittency, hierarchy, nonlinear dynamics and interactions over a wide range of spatial and temporal scales [1,2,3,4,5]. However, a simple phenomenology seems to apply to their collective behavior. The most prominent property is scale-invariance. This applies to a variety of key attributes of seismicity manifested as power–law distributions [6,7,8], as the distribution of fault-trace lengths [9], the Gutenberg–Richter scaling relation that resembles power–law scaling in the frequency of dissipative seismic energies [7] or the Omori–Utsu relation for the power–law decay rate of aftershocks [10]. Such properties motivate the statistical physics approach to fracturing and earthquakes as a consistent and promising theoretical framework for deriving the macroscopic properties observed in fault and earthquake populations from the specification of the laws that govern friction, fluid–rock interactions, fracture nucleation, propagation and so on, at the microscopic level [11]. 

Since the 1980s, when concepts such as fractals, entropy and self-organized criticality (SOC) became relevant to seismicity, considerable progress has been made in the statistical physics of earthquakes. Within this context, earthquakes are considered a critical-point phenomenon undergoing continuous phase transition [8]. According to SOC, the Earth’s crust spontaneously self-organizes in a dynamical stationary state to generate earthquakes with self-similar size distributions and fractal geometries [12]. Earthquakes occur on a fractal set of faults, characterized by long-range correlations and scale-invariant properties in their size and spatiotemporal organization [6,7,8,9]. Moreover, based on the maximum entropy principle, classic and generalized statistical mechanics can be used to infer the macroscopic properties of fractures and earthquakes from the specification of their microscopic constituents and their interactions [13]. Other statistical-physics-based models and analysis techniques that have been applied to understand the multiscale dynamics of earthquakes include renormalization group theory, phase diagrams, stochastic models, cellular automata models, correlation lengths, turbulence, percolation and fiber models, multifractals, damage mechanics models, random walks and wavelets and network theory, among others [2,3,7,8,14,15,16,17].

Some of these concepts and tools have been applied to the articles found in this Special Issue. This collection features original studies on regional seismicity that evolves into large and destructive earthquakes, as with the recent cases of the 2023 M_w_ 7.8 and M_w_ 7.6 doublet that struck the Kahramanmaraş region in East Turkey (contribution 1), the 2019 M_w_ 7.1 Ridgecrest earthquake in California (contribution 2) and the large subduction earthquakes of magnitudes greater than 7 that occurred on the Cocos subducting plate in Mexico over the last years (contribution 3). The development of early warning systems is exceptionally important in managing such extreme seismic risks, as pointed out by Donciu et al. (contribution 4) in their uniaxial shaking table testing regarding seismic frequency response. To effectively mitigate seismic risk, pattern recognition and probabilistic forecasting of earthquake occurrence using appropriate statistical models are essential, as discussed by Varini and Rotondi (contribution 5) in relation to large earthquakes in Italy and by Anyfadi et al. (contribution 6) regarding major subduction zone aftershock sequences. In this vein, emerging machine learning approaches, such as the NESTORE algorithm applied to the seismicity of Greece by Anyfadi et al. (contribution 7), are becoming more and more valuable. Nonetheless, the theoretical comprehensiveness of fundamental empirical scaling relations in observational seismology, such as the Omori–Utsu relation of the aftershock production rate discussed by Abe et al. (contribution 8) and the development of models that can mimic the physical mechanisms of earthquakes (contribution 9), are important in better understanding earthquake interactions and evolution. In addition, complex network approaches to the physics of earthquakes, as applied to intraplate seismicity in Norway by Pavez-Orrego and Pastén (contribution 10), have been in constant development in recent times. Finally, the integrative study of other natural complex systems with earthquakes, such as solar flare fluctuations, as discussed by Morikawa and Nakamichi (contribution 11), may provide universal patterns regarding the physical behavior of such systems.

Despite the considerable progress that has been achieved over the last forty years, fundamental challenges regarding the complexity and the statistical physics of earthquakes remain wide open, with many important findings anticipated in the years to come. Not only do the exact dynamics that lead to the deformation of the Earth’s brittle crust and the subsequent generation of earthquakes remain unknown, but the physical laws that govern friction, rheological and chemical processes, as well as fracture nucleation and propagation at a microscopic scale, are generally elusive and at a primal stage [18,19]. Statistical physics thus remains an expedient framework for bridging the gap between the complex microscopic laws that govern the deformation and brittle failure of solid earth materials and the macroscopic behavior of their ensemble average manifested in fault networks and regional seismicity [11,13,19,20]. Given the overwhelming amount of data that are continually collected, the constantly increasing computational power available and the new models and artificial intelligence methods that emerge, statistical physics, in synergy with seismology and other related fields branching from geology and physics, can lead to a unified framework that will provide a better understanding of the earthquake generation phenomenon, with the ultimate goal of providing efficient earthquake forecasting that can effectively mitigate risk for people and infrastructures.
